# Experience-dependent shifts in behavioural decision-making following repeated captures in a wild mammal

**DOI:** 10.1098/rsos.260223

**Published:** 2026-10-21

**Authors:** Fragkiskos Darmis, Yiannis G. Zevgolis

**Affiliations:** ^1^ Research Group Behavioural Ecology of Individual Differences, Max Planck Institute for Evolutionary Biology, 24306, Plön, Germany; ^2^ Biodiversity Conservation Laboratory, Department of Environment, University of the Aegean, Mitilini, Greece

**Keywords:** boldness, trapping, capture–recapture, exploration, personality, rodents

## Abstract

Live trapping is central to behavioural ecology, yet whether post-capture behaviour reflects consistent among-individual differences and how it is modified by repeated captures remain poorly understood. Here, we repeatedly captured wild individuals of the rodent *Apodemus mystacinus* and temporarily held them in a standardised wooden experimental box. We then allowed them to voluntarily exit the box near the capture location and continuously recorded their behaviour for 5 minutes. Using multilevel, multivariate and continuous-time multi-state models, we characterise the sequence of behavioural states from confinement to escape. We also test whether capture experience reorganises individual behavioural trajectories and whether individual characteristics, such as body mass and sex, mediate behavioural transitions across capture–recapture events. We show that there is among-individual variation in the measured post-capture behaviours and that repeated captures reorganise the structure and sequential organisation of behavioural responses after individuals exit a trap. Importantly, we also show that recapture experience and body mass are associated with an increased tendency towards rapid escape. In contrast, we find that sex has no detectable effects. Our findings highlight that behaviours expressed immediately after trap release do not represent individuals' undisturbed ecological baseline, but instead experience-dependent responses shaped by individuals' cumulative history of interactions with their environment.

## 1. Introduction

Animals respond to external stressors through complex decision-making. For example, coping with changes in food availability, quality or predation pressure [1–3] requires behavioural adjustments [4–8] that depend on evolved cue–response systems, the reliability of environmental information and behavioural flexibility [9–11], the latter defined as the capacity to adjust behaviour to new conditions.

Behavioural flexibility is considered advantageous across taxa because it promotes adaptation to novel environments and predicts invasiveness [9,12,13]. However, behavioural responses are also determined by personality [14–19]/behavioural type [20], that is; consistent among-individual variation in behaviour across contexts and time. Indeed, some individuals are consistently bolder or more aggressive than conspecifics [21–23]. Importantly, these consistent behavioural differences and behavioural flexibility are not mutually exclusive: individuals may adjust behaviour according to a stimulus while still maintaining consistent differences relative to one another [24–29]. Such behavioural variation is often associated with fitness consequences [30–35] and may therefore be the target of selection.

Behavioural responses are not only shaped by flexibility and consistent behavioural differences among individuals, but also by previous experience: repeated exposure to a situation can alter behaviour through processes such as habituation, a decline in response with repeated, non-reinforced exposure, or sensitisation, an increase in responsiveness with repeated exposure [36]. Because live trapping is a fundamental tool in ecological and behavioural research, and is commonly applied repeatedly to the same individuals, capture-recapture studies provide a particularly relevant context for examining such experience-dependent behavioural flexibility. For example, in pied flycatchers (*Ficedula hypoleuca*), repeated capture events reduce the likelihood that individuals subsequently enter nest traps, indicating long-term changes in trappability consistent with a learned avoidance of the trapping event [37]. Across taxa, such experience-dependent processes allow individuals to adjust their behaviour based on repeated interactions with their environment [30–34,37]. However, whether these adjustments reinforce, modify or obscure consistent among-individual behavioural differences and post-capture behavioural responses in wild populations remains largely unresolved.

Addressing this gap is challenging because standardised laboratory assays that have been instrumental in linking behavioural and ecological research, such as open-field tests [35,38], may not fully capture the dynamic nature of behaviour expressed in the field [39]. This is because they are often conducted under artificial light, odours and sounds that can themselves induce stress responses. As a result, they may fail to disentangle stress-related behaviour that would also be expressed under ecologically relevant conditions from that elicited by the stressfulness of the experimental design. Studying behaviour in natural contexts is therefore essential for understanding how consistent behavioural differences among individuals, as well as flexibility and experience, interact to shape responses to environmental stressors.

Live trapping provides a unique opportunity to examine this interplay because it represents a standardised, acute stressor that elicits a suite of behavioural responses among individuals. Among others, behaviour immediately following release may reflect the combined (and by extension difficult to disentangle) effects of personality (i.e. behavioural differences among individuals), behavioural flexibility and previous capture experience [30]. Indeed, several studies show that personality affects trapping or recapture probability [31,40,41], as for example in the multimammate mice (*Mastomys natalensis*), where sensitivity to stress is negatively associated with recapture probability [32]. Consequently, if consistent behavioural differences among individuals (e.g. exploration tendencies) influence movement and space use [42], then trappability should be inherently linked to these behavioural differences. At the same time, however, other empirical studies report no significant relationship between such behavioural differences and trapping probability [33,34]. One important consideration in these studies is that, because live-trapping can itself induce stress, unequal trap-induced stress among individuals or capture events could bias estimates of variability in behavioural responses; hence, this could confound behavioural measurements if not explicitly accounted for [43]. This concern is not merely theoretical, as capture history has the potential to alter behavioural readouts [31,41]. Resolving this requires moving beyond capture probability alone and explicitly quantifying behavioural responses immediately following capture and release across recapture events to disentangle experience effects from consistent individual differences in behaviour.

This emphasis on immediate post-capture behaviour also requires caution, because capture, handling and tagging can alter behaviour and movement after release, including short-term hypoactivity, hyperactivity, altered movement rates and variable recovery times [44–48]. These effects have been documented across taxa and study systems, including ungulates, wild boar and GPS-collared mammals, highlighting that behaviour recorded immediately after capture should not be interpreted as equivalent to undisturbed baseline behaviour. However, standardised post-capture assays can still provide useful information when all individuals experience the same handling, holding and release context because they allow consistent comparison of behavioural responses among individuals and across repeated capture events.

Here, we approach live trapping as a standardised, ecologically relevant stressor that allows us to probe how experience (i.e. repeated captures) and intrinsic traits, such as body mass, shape behavioural decision-making under stress. We focus on the behaviour of wild-caught Eastern broad-toothed field mice (*Apodemus mystacinus*), a murid rodent that is relatively common and often dominant within small mammal communities of the northeastern Mediterranean [49]. Despite its ecological prominence, this species has received almost no representation in behavioural studies, with most work in the region focusing on other congeners such as *Apodemus sylvaticus* [50,51].

To capitalise on this potential, we designed a field experiment in which we first live-trapped individuals and quantified their behaviour under release conditions. Specifically, we quantified behaviour following live-trapping, a stressful situation for most animals [30,52,53], by allowing individuals to voluntarily exit a standardised holding–release chamber (SHRC; ‘box’ hereafter; supplementary material, figure S1) under minimal human presence and standardised release conditions. We measured both the probability of exiting and latency to exit, as well as the sequence of behaviours expressed after exit. By repeating this procedure across capture sessions for the same individuals, we incorporated recapture number and could evaluate experience-dependent behavioural changes while also accounting for consistent among-individual differences. Importantly, because theory predicts that behavioural decisions can vary with energetic condition, we also considered body mass and sex as potential drivers of behavioural variation. Body mass is commonly used as a proxy for condition/energetic state [3,54], and individuals in different conditions may differ in their willingness to take risks or approach a potentially threatening situation [55,56]. Likewise, males and females may differ in behavioural responses owing to differences in life-history strategies and energetic demands.

Using the videos, we scored whether each mouse exited the box, and the latency to exit it, as well as the time spent in three behavioural states: (1) inside the box, (2) outside actively exploring and (3) outside frozen/stationary. We also recorded whether and when individuals left the release area by running away (4), which we treated as an event (an absorbing state) rather than a duration (see table 1). We then quantified time allocation among behavioural states and transition rates, estimated consistent among-individual variation in behaviour across captures and tested how recapture number and body mass influenced both time allocation among behavioural states and transition dynamics. In addition, we used a multivariate approach (Principal Component Analysis) to assess whether the overall structure of behavioural variation, that is; how these behavioural states co-vary, shifted between first captures and recaptures, providing a complementary, integrative perspective on how experience shapes behavioural organisation. Consequently, here we answer whether:

— repeated captures produced experience-dependent behavioural shifts;
— post-capture behaviours were consistently different among individuals; and
— body mass or sex modulated these patterns.

## 2. Material and Methods

### 2.1. Study sites and species selection

Fieldwork was conducted on the island of Lesvos, situated in the north-eastern Aegean Sea, Greece. The island covers 1632.8 km² and is characterised by a typical Mediterranean climate, with hot, dry summers and cool, wet winters. Lesvos supports a diverse terrestrial mammal fauna comprising 15 species of small terrestrial mammals, including 10 rodent species (Rodentia), four insectivore species (Eulipotyphla) and one lagomorph (Lagomorpha), alongside several carnivores and bats. Among rodents, the Eastern broad-toothed field mouse (*A. mystacinus*) is both widespread and abundant, often dominating small mammal assemblages in forested, shrub-covered and rocky habitats [49].

We selected two study sites located within mature pine-dominated forests in the central part of the island, separated by approximately 7 km (figure 1). Sites were chosen based on three criteria: (i) confirmed high occurrence of *A. mystacinus* from pilot trapping surveys, (ii) structurally complex understory and ground cover suitable for natural escape and exploratory behaviour and (iii) complete absence of human disturbance. The two sites shared similar vegetation structure and elevation, minimising environmental heterogeneity while allowing replication across spatially distinct populations.

**Figure 1.**
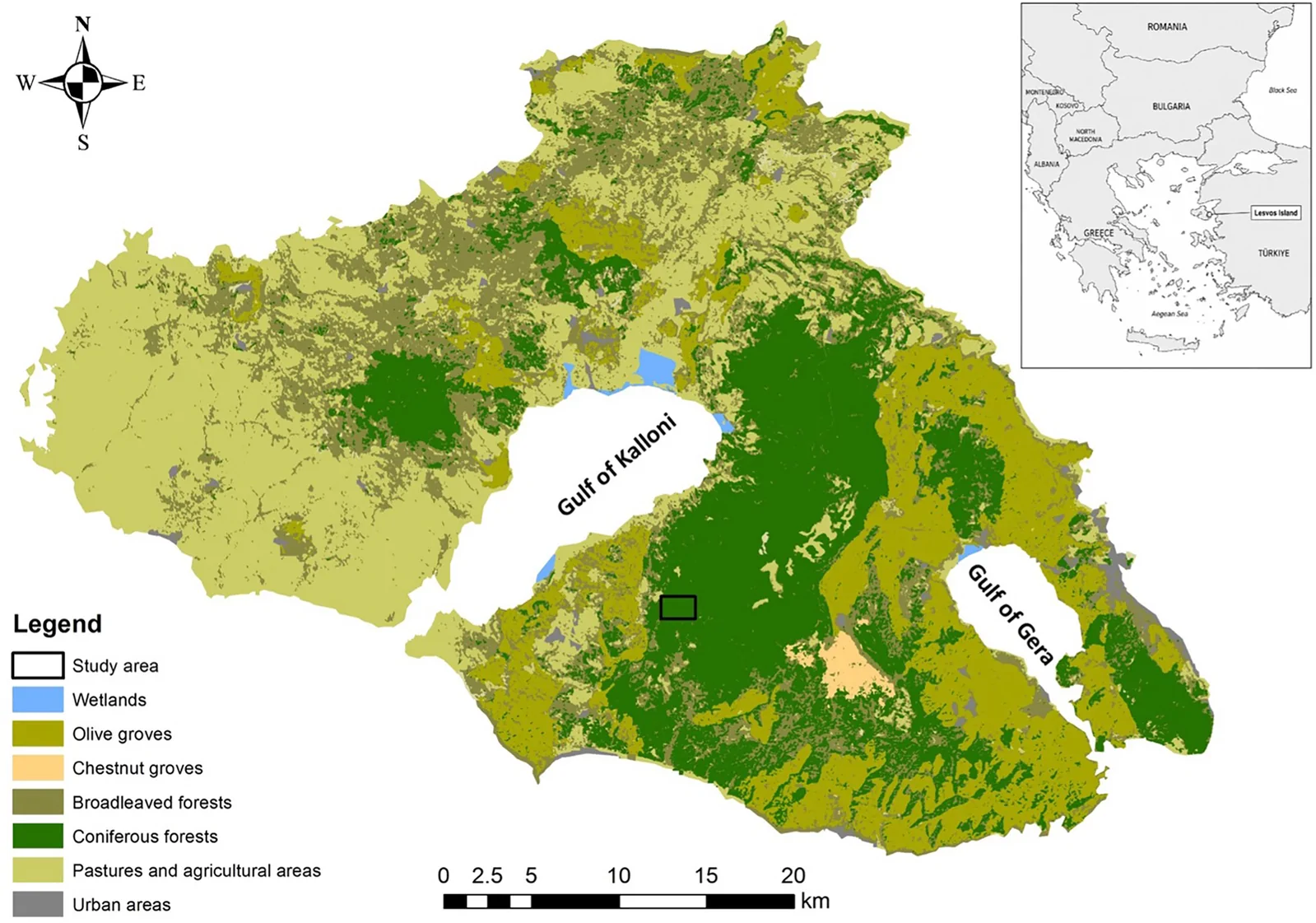
Study area on the island of Lesvos, north-eastern Aegean Sea, Greece. Land cover types are shown, including wetlands, olive groves, chestnut groves, broadleaved forests, coniferous forests, pastures and agricultural areas and urban settlements. The black rectangle indicates the locations of the two trapping sites where Eastern broad-toothed field mice (*A. mystacinus*) were sampled. The inset map shows the location of Lesvos within the north-eastern Aegean and south-eastern Europe.

### 2.2. Trapping procedure

Trapping was conducted in 2020 during two seasonal periods: spring (April–May) and autumn (September–November). At each site, 20 collapsible Sherman live-traps (LFATDG, 7.6 × 8.9 × 22.9 cm; H.B. Sherman Traps Inc.) were deployed nightly along linear transects. Traps were spaced at regular intervals (approximately 7–10 m, depending on terrain) and placed in microhabitats typically used by *A. mystacinus*, such as along runways, near ground cover and at the base of shrubs or fallen logs. Each trapping session comprised five consecutive nights, followed by a one-week interval during which no trapping occurred, and was then repeated for an additional five nights. Traps were baited with a standardised amount of peanut butter (approximately equal to 5 g), which was used consistently across sites and capture events, and bait was replaced daily. In all capture events, the bait had been fully consumed by the captured individual by the time traps were checked, and traps were cleaned between uses to ensure that residual odour from previous animals did not affect the behaviour of subsequently trapped individuals. Traps were set approximately 30 minutes before sunset and inspected at dawn, with all checks completed within one hour of sunrise to minimise confinement time and associated stress. To improve animal welfare and ensure reliable captures, traps were insulated with bubble wrap and lined with locally collected dry grass and pine needles, which reduced heat loss and provided bedding material.

Across both sites, total trapping effort amounted to 800 trap-nights, yielding 93 capture events for 27 individuals, with 27 first captures and 66 recaptures. All captured individuals were adults, as determined by body size and reproductive characteristics, ensuring that behavioural comparisons were not confounded by ontogenetic stage. Any non-target captures were released immediately at the point of capture.

The temporal spacing between capture events within individuals was heterogeneous and right-skewed. Across all inter-capture intervals among recaptured individuals (*n* = 66), the mean interval was 22 ± 35 days, the median interval was 13 days, and the range was 1–157 days. Most intervals occurred within two weeks (58/66 intervals; 88%), whereas six extended intervals ranged from 138 to 157 days. Only three individuals were recaptured across both seasonal periods, and six individuals were captured only once.

### 2.3. Handling and biometric measurements

Upon capture, individuals were carefully removed from traps and subjected to a standardised biometric protocol. Body mass was measured to the nearest 0.1 g using a mechanical spring balance (Pesola AG, Switzerland). Mean body mass of captured individuals was 36.9 ± 7.42 g (range: 24.4–59 g) and did not differ detectably between first captures and recaptures when accounting for repeated measurements of the same individuals. Sex was determined by genital morphology (N_females_ = 11; N_males_ = 16). First-captured individuals were fitted with numbered metal ear tags for individual recognition; tag placement required a small puncture of the ear tissue. To standardise handling across first captures and recaptures, recaptured individuals were subjected to the same handling sequence and duration, including inspection of the ears and existing tag and simulation of the ear-tagging manipulation, but no additional puncture or new tag was applied. The entire handling process was completed in less than five minutes per animal to minimise stress.

### 2.4. Release protocol

Following handling, each individual was placed in the box (supplementary material, figure S1), which was designed to minimise immediate handling-related stress and to provide a standardised environment for subsequent behavioural observations. The box was a fully enclosed wooden chamber with a square base (40 × 40 cm) and a height of 50 cm. All walls and the roof were opaque, creating near-total darkness inside the chamber. This design eliminated external visual cues and standardised sensory conditions across individuals and capture events, thereby reducing context-dependent behavioural bias.

The floor of the box was lined with freshly collected dry grass and leaves, providing a familiar substrate and thermal buffering. Substrate material was replaced between trials to prevent odour accumulation and chemical cue transfer among individuals. Each mouse remained inside the box for 5 minutes. This holding period was selected based on previous field experience and pilot observations as a compromise between allowing behavioural stabilisation following handling and minimising confinement duration, thereby reducing stress-related artefacts while maintaining consistency across trials. At the end of the holding period, four square exit openings (5 × 5 cm), positioned centrally on each side wall of the box, were opened simultaneously using a manual rope-release system. All exits becoming available at the same moment prevented directional or sequential bias in exit choice. The release mechanism was operated from several metres to minimise human presence and disturbance at release.

The box was placed within the same *Pinus brutia* forest habitat where individuals were captured. Thus, mice exited into a relatively sheltered natural forest environment rather than an open or brightly exposed area. The immediate release area consisted of natural forest-floor substrate, including pine needles, dry grass, leaf litter and nearby understory/ground cover, providing familiar refuge and escape structures comparable to the habitat used by the animals at the trapping sites.

Individuals were not forced or encouraged to exit the box. Mice could remain inside the box or leave voluntarily, allowing exit decisions and latencies to reflect spontaneous behaviour rather than induced movement. Behaviour, while the individual remained within the box, was operationally defined as the ‘inside’ state, whereas post-exit behaviour was recorded only after the individual had fully exited the box with all four paws outside. Behavioural monitoring was conducted using two synchronised video cameras positioned externally on opposite sides of the box, each approximately 5 m away. Each camera was oriented to record two of the four exit openings; together, the two cameras provided continuous visual coverage of all four exits and the surrounding release area within an approximately 5 m radius of the box, corresponding to ca. 80 m². Camera placement and fields of view were adjusted before data collection to ensure continuous visual coverage of all four exits and the immediate surrounding release area, with no blind zones in which exit events or initial post-exit behaviours could occur unrecorded. Because the interior of the box was intentionally dark and visually isolated, behaviour within the box was quantified as presence inside versus exit, rather than through fine-scale internal movement, thereby avoiding observer bias associated with partial or uneven visibility.

Each release was recorded continuously for 5 minutes following door opening. This standardised observation window allowed consistent quantification of exit decisions and early post-exit behavioural sequences across individuals and capture events under standardised low-interference field conditions.

### 2.5. Behavioural state definitions and transition coding

Video recordings were analysed using BORIS by a single observer. We quantified behavioural states following box opening, for up to 5 minutes, by defining four mutually exclusive behavioural states (table 1): (1) inside the box, when the individual remained fully inside the box (all four paws inside); (2) outside actively exploring, when the individual was outside the box (all four paws outside) and engaged in continuous locomotion; (3) outside stationary (frozen), when the individual was outside the box (all four paws outside) but immobile; and (4) running away, defined as rapid, directed movement leading the individual to leave the release area. For each individual and capture event, we quantified the time spent in states 1–3. Time after an individual left the release area by running away was not scored; therefore, the summed time in states 1–3 is less than 300 s in trials where running occurred. In addition, we recorded whether individuals exited the box and measured the latency to exit (the time from door opening until the individual first exited the box with all four paws).

**Table 1.** Ethogram and derived behavioural measures scored from video recordings for 5 minutes after door opening. ^1^Behavioural states represent mutually exclusive categories describing the temporal sequence of behaviour, from which derived behavioural measures were calculated. Time spent in states 1–3 (inside the box, outside actively exploring, outside frozen/stationary) was quantified as a duration (in seconds) for each capture event. ‘Running away’ (state 4) was not scored as a duration; it was recorded as an event, and treated as an absorbing state in the multi-state model, such that no further behavioural transitions were recorded for that trial once an individual had left the release area.

types of behaviours	variable	description
behavioural measures	exited the box	binary variable (yes/no) indicating whether the individual exited the box within 5 minutes of door opening
	time to exit the box	time from door opening until the individual first exited the box (all four paws outside); individuals that did not leave the box within 5 minutes of door opening were treated as right censored
behavioural states^1^	time inside the box	individual remains fully inside the box (all four paws inside)
	time outside the box actively exploring	individual is outside the box (all four paws outside) and engages in continuous locomotion
	time outside the box frozen/stationary	individual is outside the box (all four paws outside) and immobile (no locomotion)
	outside the box running away from the release area	individual leaves the release area via rapid, directed movement

To investigate behavioural transitions and the time spent in each state (multi-state model; see below), we ordered observations by individual identity (ID), recapture number and the sequence of behavioural records within each capture. For each record, we defined the current behavioural state as the ‘from’ state and the next observed behavioural state as the ‘to’ state. For example, if an individual inside the box exited and started exploring, this transition was coded as inside → outside exploring. We retained recapture number as a numeric covariate (recapture) and coded behavioural states as categorical variables with the following ordered levels: inside, outside exploring, outside frozen and running.

### 2.6. Statistical analyses

All statistical analyses were conducted in R (version 4.4.1, 2024-06-14) and all models were run in a frequentist framework using *lme4* [57]. Validity of all models was assessed visually by inspecting the distribution of residuals and by calculating the variance inflation factor using the *performance* package [58], with no predictors being highly correlated. All continuous covariates were standardised (mean = 0, s.d. = 1) to improve model convergence and interpretability.

We determined the proportion of variance in behaviours that was attributable to among-individual differences (i.e. personality) by calculating the (adjusted) repeatability using the *rptR* package [59]. All models were fitted with bootstrapping and permutation (1000 replicates for both). Repeatability measures the proportion of total phenotypic variance attributable to differences among individuals, with behavioural traits such as boldness and stress-coping showing, on average, approximately 37% repeatability [60]. As we were interested in determining if behaviour is consistently different among individuals by controlling for the effect of recapture number, we fitted three models: first, we modelled whether a mouse exited the box as a binary response (model 1; see table 2), including individual identity (ID) as a random effect, sex, body mass and recapture number as fixed effects; second, we modelled the latency to exit the box (model 2) using a Cox proportional hazards model (coxme package), treating latencies (i.e. time taken to exit) for individuals than never exited as censored. Repeatability was then computed following the approach of McCune *et al.* [61], and the model included the same fixed and random-effect structure as model 1. Last (model 3), we fitted one Gaussian model with the time spent in behavioural states (inside the box, outside the box exploring or outside the box being frozen/stationary) as a response, including individual identity as a random effect and recapture number, body mass, sex and state as fixed effects. Thus, in model 3, each individual during each capture event contributed multiple rows, one for the time in each behavioural state (e.g. inside, frozen outside, exploring outside; ‘running away’ state, by definition, had no value). While we acknowledge that these behavioural states likely represent distinct dimensions related to personality, and could therefore be modelled independently, we used one model (model 3) for three reasons. First, including behavioural state as a fixed effect accounts for systematic differences among states. Second, because these behaviours were measured within a fixed 5-minute observation window, they represent components of a constrained time budget rather than fully independent behavioural dimensions. Third, sample sizes for some states were limited, which precluded reliable estimation of repeatability in separate models.

**Table 2.** Overview of statistical approaches used. See §2.6 for details on model structure and the supplementary tables for full model outputs. Random effects are in *italics*.

type of analysis and response variable	structure
model 1: repeatability of the probability of exiting the box^1^	sex + body mass + recapture number + *ID*
model 2: repeatability of the latency to exit the box^1^	sex + body mass + recapture number + *ID*
model 3: repeatability of the time spent in each state^1^	sex + body mass + recapture number + state + *ID*
model 4 (GLMM^2^): probability of exiting the box	sex + recapture number + body mass + *ID*
model 5 (Cox^3^): time to exit the box	sex + recapture number + body mass + *ID*
model 6 (LMM^2^): time spent in each state after exiting the box	sex + recapture number + state + body mass + *ID*
model 7 (MSM^4^): transitions between behavioural states^6^	recapture number + body mass + *ID*
PCA^5^ 1: first captures	^6^time inside the box + time to exit the box + time outside exploring + time outside frozen
PCA^5^ 2: recaptures	^6^time inside the box + time to exit the box + time outside exploring + time outside frozen

^1^Repeatability analysis using *rptR*. ^2^(Generalised) linear mixed-effects model using *lme4*. ^3^Cox proportional hazards model. ^4^Multi-state model using *msm*. ^5^Principal component analysis. ^6^For a clear description of these variables (i.e. states), how they were measured and their biological significance, see §2.5 and supplementary material, table S3.

We then fitted corresponding models to test whether exiting the box (yes/no response; model 4), the time taken to exit the box (Cox model; model 5), and the time spent in each behavioural state (model 6: inside, frozen outside, exploring outside) were influenced by recapture number, body mass and sex. While models 4–6 use the same response variables and essentially the same fixed- and random-effect structures as models 1–3, they serve a different inferential purpose: models 1–3 partition variance to estimate adjusted repeatability, whereas models 4–6 estimate and test whether specific fixed effects, including recapture number, body mass and sex, predict variation in the corresponding behavioural responses. Indeed, we included sex and body mass as fixed effects to test for sex-specific effects and whether variation in body condition explained variation in behaviour, respectively. For model 5, we treated individuals that did not exit the box within the 5-minute window as censored. As previously, each individual during each capture event contributed multiple rows in model 6, one for the time in each behavioural state. Because of this, in model 6 we controlled for behavioural state (time ∼ state + sex+ body mass + recapture number + ID; see table 2). Individual identity was included as a random intercept in all models to account for repeated recaptures of the same individuals.

We ran one continuous-time multi-state Markov model using the *msm* package [62] to estimate transition intensities, that is; the instantaneous rates of moving from one state to another, and allow the inclusion of covariates affecting these transitions. We assumed that transitions between behavioural states depended only on the current state (i.e. are memoryless) and that transition rates remain constant over the 5-minute observation period. The transition intensity matrix (*Q*) was specified such that all off-diagonal transitions between states were allowed (except those out of “running away”, the absorbing state). This method allowed us to analyse the sequential behavioural responses of individual mice during first captures and recaptures (model 7). In the model, we fitted as a response variable one of four mutually exclusive behavioural states: inside the box, outside exploring, outside frozen and running away. As covariates, we included individual weight measured at each capture and recapture number (first capture vs recapture). Because sex was not associated with any behavioural measure in the preceding analyses, and its inclusion did not improve model fit (not shown), we excluded it from the final Markov model. Importantly, to conduct this analysis, we constructed a cumulative observation sequence across multiple captures per individual, ensuring a strictly increasing time variable. Individuals with only a single behavioural observation were excluded because they provided no information on transitions across repeated captures. Overall, our model estimated the probability an individual is in state *j* at step *t* given that this individual was at state *i* at step *t−1*, identifying as such the behavioural sequence of behaviours individuals followed as a function of their previous behaviour, recapture number and weight. Transition probabilities for each state were estimated with 95% confidence intervals. Convergence of the model was verified using the optimiser convergence code. Transition probabilities for different covariate scenarios were obtained with *pmatrix.msm*.

Lastly, we used an unrotated principal component analysis (PCA; *prcomp* function of the stats package) using the four behavioural measures recorded during trap sessions to summarise the behavioural variation of individuals into major axes (table 2). Data were split into first captures (*n* = 27) and recaptures (*n* = 66). Individual PCA scores were extracted for visualisation and biplots were subsequently generated using the *fviz_pca_biplot* function. Individuals that never exited the box (censored) were considered with a time to exit = 5 minutes for this analysis.

**Figure 2.**
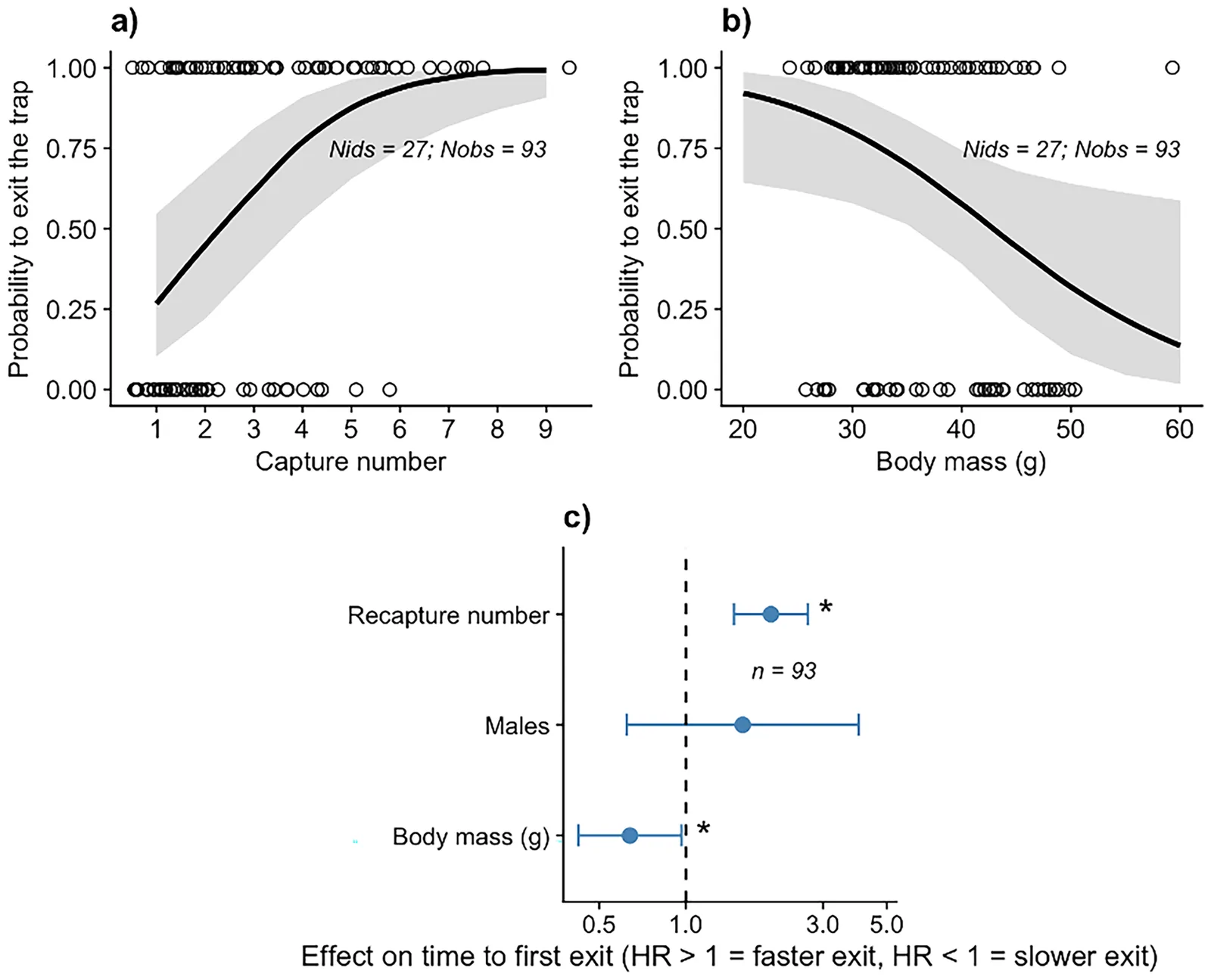
Effects of recapture number and body mass on: a) the predicted probability to exit the trap box as a function of the recapture number (experience) conditioned on sex and weight. Points represent raw data (jittered); the line represents the model prediction with a 95% confidence interval (shaded); b) the predicted probability of exiting the trap relative to the animal's weight (g), conditioned on recapture number and sex; c) forest plot showing hazard ratios (HR) extracted from a Cox proportional hazards model estimating the latency to exit the box for the first time. Hazard ratios > 1 indicate a faster exit, while < 1 indicate a slower exit. Error bars represent 95% confidence intervals; the dashed line at 1 indicates no effect. Significant effects are indicated with a *. Sample sizes (*n*) are given.

## 3. Results

In total, we had 93 capture events from 27 unique individuals, with recapture number ranging from 1 to 9, giving an average of 3.44 ± 2.26 captures per mouse. On average, mice spent 172.0 ± 126.0 seconds inside the box (range = 1–300 seconds) and considerably less time outside the box (24.0 ± 54.0 seconds, range = 0–280 seconds). Out of the 27 individuals captured at least once, 20 (approx. 74%) exited the box at least once. Of the time spent outside, active exploration was low (7.0 ± 11.0 s, range = 0–53 seconds), as was time spent stationary/frozen (17.0 ± 51.0 seconds, range = 0–275 seconds). Latency to exit the box also varied widely across individuals (165 ± 129 seconds, range = 2–300 seconds).

Repeatability analyses revealed consistent among-individual differences in all behavioural measures after accounting for recapture number (supplementary material, table S1). Specifically, the probability of exiting the box showed a marginally non-significant repeatability (*R* = 0.27, 95% CI = 0.0–0.4, *p* = 0.052), while the latency to exit (*R* = 0.29, 95% CI = 0.04–0.59, *p* < 0.05) and the time allocated across behavioural states (*R* = 0.25, 95% CI = 0.12–0.31, *p* < 0.001) reached statistical significance.

Recapture number and body mass significantly influenced behaviour. The probability of exiting increased with recapture number (log-odds est. = 1.43, 95% CI = 0.56–2.29, *p* = 0.001; figure 2a; supplementary material, table S2), but heavier individuals were less likely to exit (est. = −0.80, 95% CI = −1.50 to −0.09, *p* = 0.026; figure 2b). Consistent with this pattern, the Cox proportional hazards model indicated that individuals exited the box faster with an increasing recapture number (est. = 0.68, 95% CI = 0.39–0.98, *p* < 0.001; figure 2c) and that heavier individuals exited later (est. = −0.45, 95% CI = −0.86 to −0.03, *p* = 0.034; figure 2c). Lastly, sex did not predict any effect.

**Figure 3.**
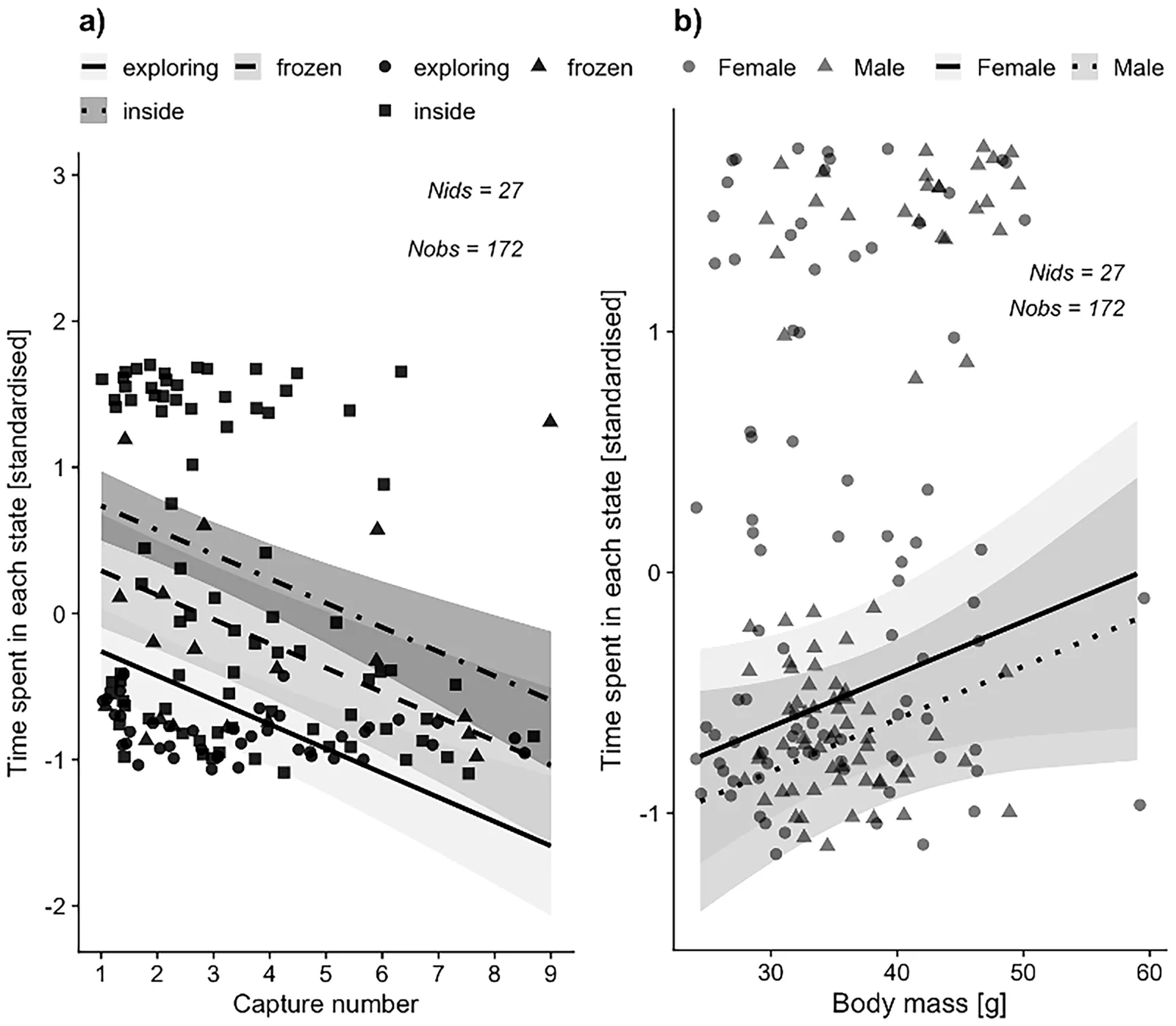
Effects of recapture number, sex and body mass on time allocation to behavioural states: (a) effect of experience (recapture number) on the standardised (mean = 0, s.d. = 1) time spent in different states (exploring outside = solid, frozen outside = dashed and inside the box = dotted). Ribbons and lines represent predicted values and 95% confidence intervals derived from the model; jittered points represent raw data observations; b) effect of body mass (g) and sex on the time spent in a state. Points represent individual observations. Sample sizes per sex (the number of observations per state) are indicated within each panel.

Individuals spent significantly more time inside the box than in outside behavioural states (est. = 1.00, 95% CI = 0.74–1.25, *p* < 0.001; figure 3a). Time allocated to behavioural states decreased significantly with increasing recapture number (est. = −0.35, 95% CI = −0.48 to −0.21, *p* < 0.001; figure 3a), indicating shorter persistence in non-running states across repeated captures. However, body mass (est. = 0.14, 95% CI = −0.02–0.31, *p* = 0.08; figure 3b) and sex did not significantly predict time allocation among states.

**Figure 4.**
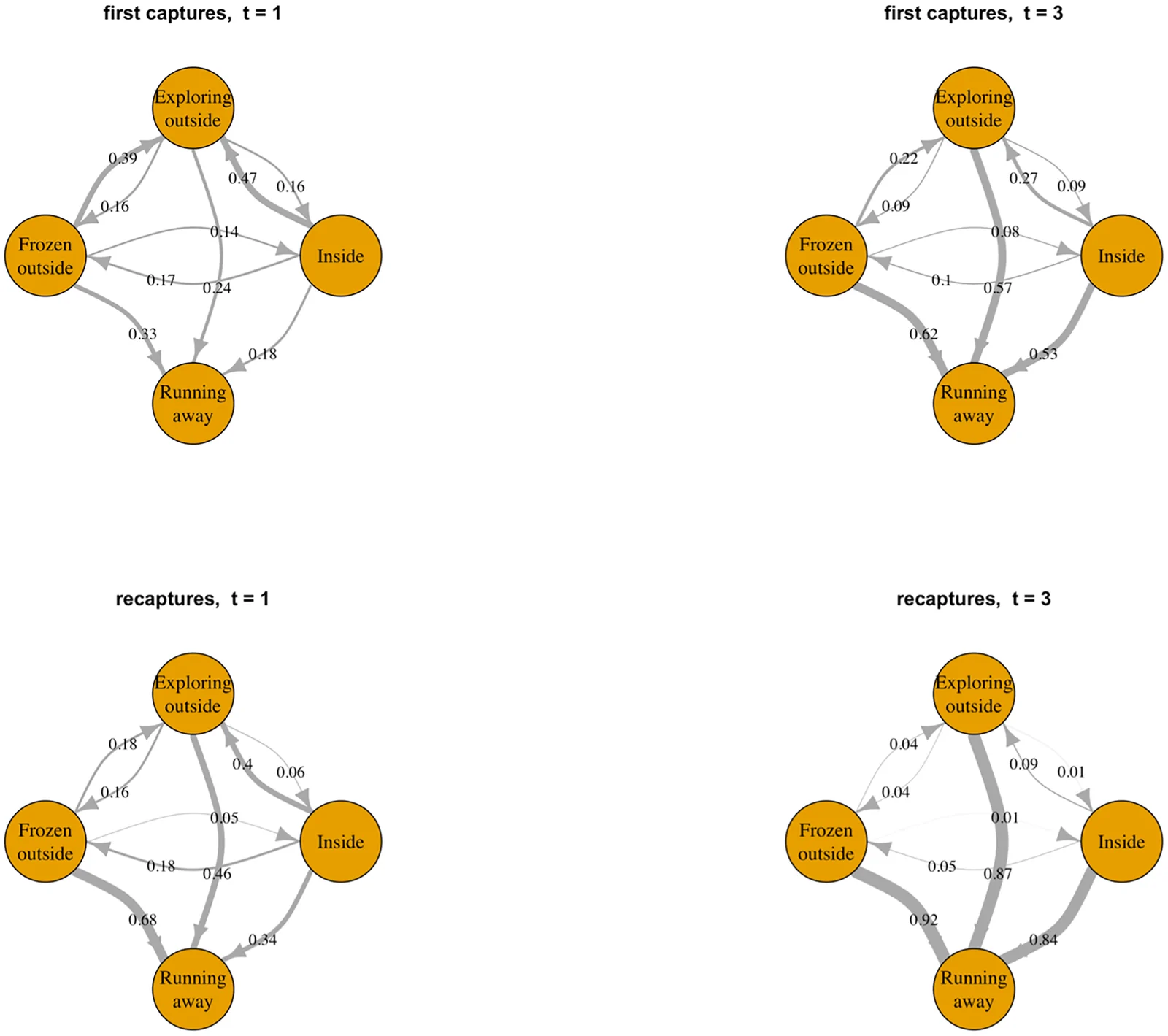
Estimated probabilities of sequential behavioural transitions for first captures and recaptures, derived from the Markov multi-state model. Each graph shows the probability of being in each behavioural state after one or three sequential transitions, conditional on the starting state. Nodes represent behavioural states and direct edges indicate allowed transitions defined by the transition matrix. Arrow widths are proportional to estimated transition probabilities, with wider arrows indicating higher probabilities. Self-loops, such as ‘frozen outside’ → ‘frozen outside,’ were excluded from the graphical representation for non-absorbing states to improve visual clarity. The state ‘running away’ was treated as absorbing within each capture event, meaning that once an individual left the release area, no further behavioural transitions were recorded for that trial. Transition probabilities are shown for the model including recapture number and body mass. Absence of an arrow corresponds to a transition probability of 0.

### 3.1. Sequential transitions and multivariate behavioural structure

While earlier models highlighted differences in time allocation among behavioural states, the Markov approach provided complementary information by explicitly characterising transitions among behavioural states following the opportunity to escape (supplementary material, table S3; figure 4). Throughout the results, one unit of model time corresponds to a single step in the observed behavioural sequence (i.e. the next recorded behavioural state within the 5-minute observation window; all transition probabilities are given in detail in supplementary material, table S3).

**Figure 5.**
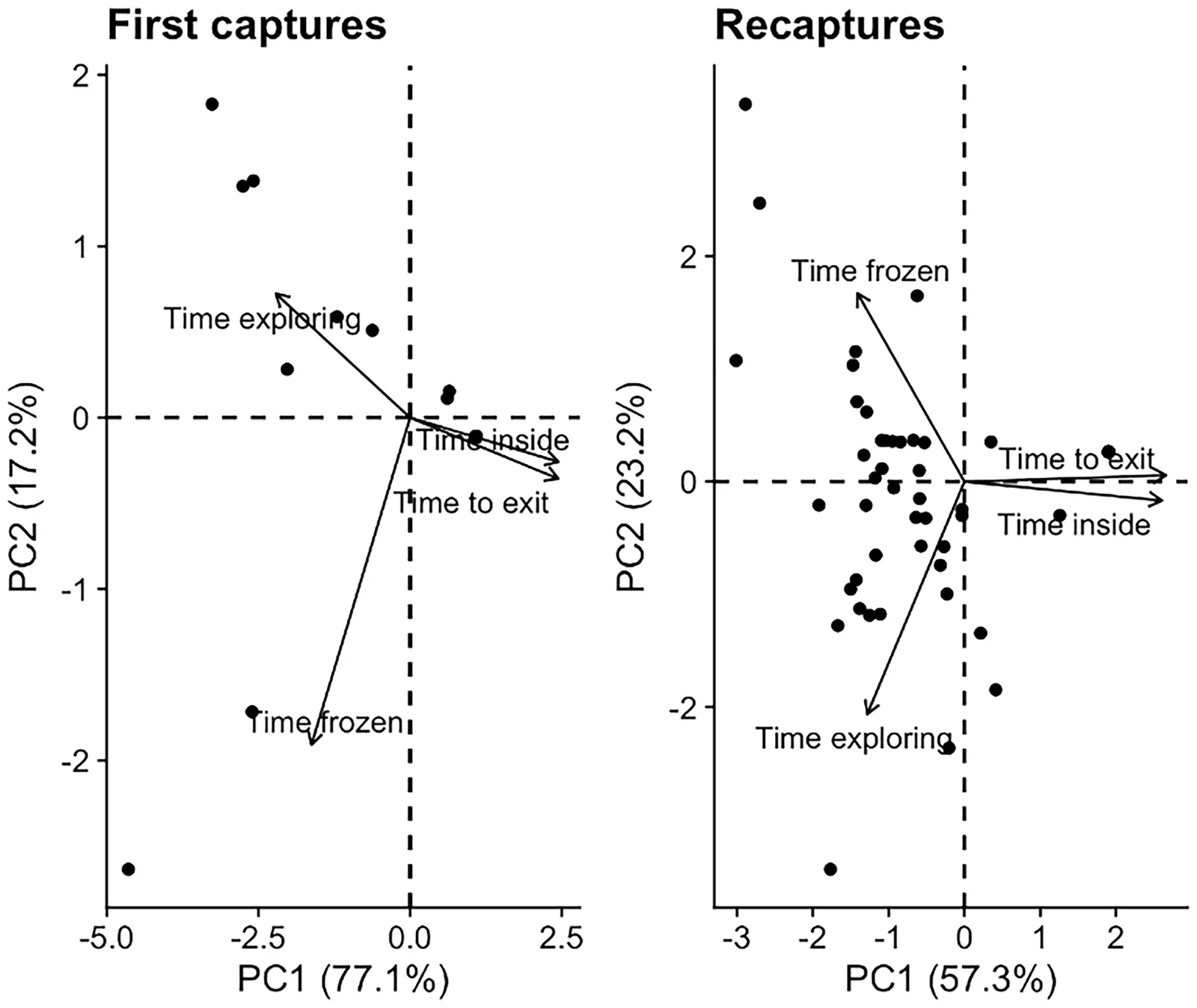
Principal component analysis (PCA) of behavioural responses during first captures (*n* = 27 observations) and recaptures (*n* = 66 observations). Points represent capture-event observations and arrows indicate the contribution of each behavioural variable to the principal components. Note that several individuals exhibited identical behavioural time budgets (e.g. remaining inside for the full 5-minute trial, corresponding to 5 minutes inside and 0 minutes in all other behavioural states), resulting in overlapping points in PCA space. Thus, the apparent reduction in the number of visible points reflects overplotting data rather than data filtering.

Body mass and capture experience strongly influenced behavioural transitions. During first captures, individuals of average or below-average body mass were most likely to transition from inside the box (i.e. inside state) to an active exploration ‘strategy’ outside the box (e.g. exploring state), while transitions to ‘frozen outside’ (i.e. exited the trap but stayed stationary) or ‘running away’ occurred with lower probabilities. Likewise, individuals that were already actively exploring or were frozen showed relatively low probabilities of immediately running away. In contrast, recaptured individuals exhibited a markedly greater tendency towards rapid escape (i.e. the running state; e.g. from 19% in first captures to 31% in recaptures), and simultaneously also decreased the probability of exploring directly after leaving the box. Similar patterns were observed for individuals already outside the box. Overall, recaptured individuals were less likely to remain frozen or explore their surroundings than first-captured ones.

During first captures, heavier-than-average individuals showed a greater tendency to transition directly to the running state (e.g. escaping) than lighter individuals, with transition probabilities that were nearly twice as high in some cases. Recapture further strengthened these patterns in heavier individuals (supplementary material, table S3): the probability of transitioning to ‘running away’ increased from all states and, simultaneously, the probability of remaining frozen declined substantially (approximately threefold), suggesting that repeated capture experience reduced hesitation and promoted rapid escape responses in heavy mice.

Recapture also substantially altered the multivariate structure of behaviour (figure 5; supplementary material, tables S4 and S5). During first captures, behavioural variation was strongly dominated by a single principal component (PC1 = 77.1%, PC2 = 17.2%, PC1 + PC2 = 94.3%). Indeed, PC1 primarily contrasted time spent inside the box and latency to exit (positive loadings = 0.55 and 0.55, respectively) with the time spent exploring and frozen outside the box (negative loadings = −0.50 and −0.37, respectively), representing a gradient from prolonged box occupancy and delayed emergence to increased activity outside the box. PC2 was driven primarily by freezing behaviour (loading = −0.91), with a weaker contribution from exploration (0.35), indicating that freezing represented an independent axis of behavioural variation during first captures. Following recapture, behavioural variation became less strongly dominated by a single axis (PC1 = 57.4%, PC2 = 23.2%, PC1 + PC2 = 80.6%), indicating a more even distribution of variance across behavioural traits. PC1 still contrasted the time spent inside the box and the latency to exit (loadings = 0.62 and 0.64) with the time spent exploring and frozen outside the box (loadings = −0.31 and −0.34), although this axis explained substantially less variation than during first captures. During recaptures, PC2 still contrasted freezing behaviour (loading = 0.63) with exploratory activity (loading = −0.78).

## 4. Discussion

In this study, we investigated the behavioural responses of wild mice *Apodemus mystacinus* in their natural environment following capture–recapture events. We first tested for consistency among-individual variation in several behaviours, after mice were free to leave the box, and assessed how these behaviours changed with successive capture events. Using a multi-state analysis, we then examined how recapture number and body mass influenced the sequence of behaviours individuals followed after being given the option to leave the box. This approach allowed us to quantify behavioural flexibility and state-switching dynamics that would not be captured by simpler analyses focused solely on behavioural outcomes. Finally, we quantified the major axes of the behavioural variation individuals displayed and how recapture number was associated with changes in these axes. Together, we provide a comprehensive view of whether recapture number, sex and body mass shape behaviour immediately after wild mice were free to leave a trap in their natural environment. Our approach therefore highlights the role of experience-dependent decision rules in the structure of consistent among-individual behavioural differences and behavioural flexibility.

Our study should be distinguished from post-capture movement studies that examine recovery, acclimation or altered movement following capture and release [44,47,48]. Such studies have shown that capture and handling can produce short-term hypoactivity, hyperactivity, altered movement rates or delayed recovery. In contrast, the box (supplementary material, figure S1) used here was designed to standardise the immediate post-capture context by providing the same holding period, release opportunity and observation window to all individuals. Thus, the behaviours measured here do not represent unrestricted baseline movement after release, but rather behavioural decisions expressed under a standardised post-capture challenge. This design allows comparison of individuals and capture histories, while longer-term tracking would be required to determine how these responses translate into natural movement or recovery after release.

### 4.1. Consistent among-individual variation in behaviour

Our findings show that *A. mystacinus* exhibited consistent among-individual differences in post-capture behavioural responses after controlling for sex, body mass and recapture number. Notably, the strongest among-individual differences emerged in the latency to exit the box, although the other measures also showed evidence for consistent among-individual variation. The estimates of repeatability we report are therefore close to the mean estimates of behavioural traits across animals [60].

In rodents and small mammals more broadly [29], behaviours related to exploration, activity and boldness are consistently different among individuals [28,63–65]. In this study, we therefore report comparable patterns for behaviours measured directly in the field that relate to risk-taking, exploratory and stress-coping tendencies. As such, our findings suggest that capture responses can be considered part of an animal's broader behavioural phenotype. Because trapping acts as an acute stressor across many animals [30,52,53], our results also posit that the consistent among-individual variation in behavioural responses under conditions similar to ours reflects a boldness–stress-coping axis, as discussed below.

### 4.2. The effect of recapture number on behaviour

We found that repeated capture was associated with changes in behaviour. Mice were more likely to exit the box, and they did so more quickly, with an increasing recapture number, revealing a pattern consistent with experience-dependent shifts arising from the capture–release procedure. This experience-dependent shift, combined with our results from the multi-state model (see below), underscores that changes in behaviour operate not only through changes in response magnitude but also through reconfiguration of behavioural decision pathways related to stress. In contrast to here, in deer mice and voles, the time spent in a box does not affect behavioural measurements of wild individuals, so whether an animal is naïve to trapping plays a role in the effects of confinement duration on behaviour [43]. Notably, the direction of the changes we report here, faster and more frequent exiting of the box as a function of recapture number, together with an increased tendency towards rapid escape (see below), is not fully consistent with habituation, which is classically defined as a decreased response to a repeated, non-threatening stimulus. Instead, this pattern may reflect sensitisation, whereby responsiveness to a repeated stressful stimulus increases with experience. Alternatively, these behavioural changes may reflect an experience-dependent shift towards a more efficient active coping strategy. Within the broader framework of habituation and sensitisation [36], our behavioural measures do not directly distinguish the underlying learning process. We therefore interpret these patterns conservatively as experience-dependent changes without assigning them specifically to either habituation or sensitisation.

Body mass also predicted behavioural responses after individuals were given the option to voluntarily leave. Consequently, variation in body mass, as a proxy for individual state or condition, contributed to behavioural variation under stress. Heavier individuals were less likely to exit the box, took longer to exit than lighter conspecifics and spent significantly more time in all states during transitions (the latter effect was at a statistical significance of *p* = 0.08). These patterns are consistent with condition-dependent stress-coping and risk-aversion, and align with the asset-protection principle [55]. Specifically, individuals in better condition (those with more ‘assets’, in our case heavier individuals) face higher potential costs in risky situations and therefore adopt more cautious strategies. Similar patterns have been reported in wild mice, where heavier individuals preferentially use low-risk rather than high-risk foraging areas [3]. A recent meta-analysis has further demonstrated a negative relationship between risk-taking behaviour and nutritional condition or quality across species [66]. Consequently, repeated capture and intrinsic condition jointly shape stress-induced behavioural decisions, combining effects of experience with condition-dependent risk aversion. Crucially, because body mass may also covary with age and reflect accumulated experience, we cannot exclude the possibility that some of these patterns are partly attributable to age-related differences in stress-coping/risk-taking behaviour rather than condition alone.

### 4.3. Behavioural flexibility after capture: transitions and behavioural structure

Our multi-state approach revealed important nuances in responses to stress as a function of previous experience and body mass when individuals were given the opportunity to exit a trap. First-capture (naive) individuals, particularly those of lower body mass, tended to prioritise environmental assessment and exploratory behaviour over rapid escape. These individuals showed lower probabilities of immediate exit and greater persistence in non-escape behavioural states, frequently switching among exploratory and stationary behaviours around the release area. In contrast, heavier individuals exhibited a more direct behavioural trajectory towards escape, with higher probabilities of transitioning rapidly to running behaviour once exiting the box. As a result, heavier individuals occupied fewer behavioural states overall. Recapture number was associated with consistent shifts in transition structure irrespective of body mass, indicating experience-dependent flexibility in decision-making.

Our findings further demonstrate that experience alters the structure of behavioural sequences rather than simply the frequency of individual behaviours. Irrespective of body mass, recaptured individuals showed an increased transition probability towards running and a reduced probability of remaining in exploratory or stationary states. However, this effect was not uniform: the increase was more pronounced in lighter individuals, possibly because heavier individuals already exhibited relatively high baseline probabilities of escape during first captures. This suggests that previous trapping experience may modify the transition probabilities governing behavioural decisions, while intrinsic traits such as body mass modulate the strength of these responses. In other words, exposure to trapping may reduce hesitation and promote faster escape responses in individuals with more active stress-coping strategies. These patterns are consistent with individual differences in baseline stress responsiveness associated with condition [66]. Indeed, smaller-bodied mammals often show stronger physiological stress responses during acute manipulations [53,67], an effect unlikely to be explained solely by thermoregulatory constraints [68,69]. It is therefore possible that lighter individuals, potentially in poorer condition, exhibit greater behavioural flexibility across capture events. Alternatively, heavier individuals may represent reproductively active or higher-ranking animals, as in mice [70], that adopt risk-averse strategies. Disentangling these alternatives will require independent measures of reproductive status, social complexity and energetic condition.

Overall, our results from the Markov model highlight that indicators of condition, such as body mass, as well as experience (here recapture number), interact to shape behavioural decision-making under stress, consistent with previous work showing that previous experience can modulate stress-coping and boldness behaviours in wild rodents [30,63,71]. A comparable effect of recapture number is also seen in mountain pygmy-possums, where newly captured animals have a lower recapture probability and take fewer risks during foraging compared to previously captured animals [72]. Collectively, the more cautious escape responses of some individuals suggest that mice in poorer condition may prioritise environmental sampling to maximise information acquisition [73,74]. In contrast, heavier individuals may adopt an asset-protection strategy [55,75], minimising such risk exposure.

The PCA further clarified how behavioural structure changes with experience by revealing a reorganisation of the covariance structure of behaviours across recapture events. At first capture, behavioural variation was strongly concentrated along a dominant axis reflecting a continuum from prolonged box occupancy to post-release activity. This axis contrasted time spent inside the box and latency to exit with time spent exploring and freezing outside the box, effectively separating individuals remaining within the box from those engaging in behaviours that consumed energy. A second, independent axis captured variation in freezing behaviour, indicating that immobility represented a distinct component of behavioural variation at initial capture. We interpret the first axis as a gradient of active versus passive stress-coping styles [4], consistent with previously described syndromes in rodents [71,76,77]. Importantly, active versus passive stress-coping behaviours are linked with stress physiology [63]: individuals that avoid central areas and stay inactive during stress (here mice that stayed inside the box) exhibit higher faecal cortisol levels, that is; risk-avoidant or shy phenotypes [25,65,78].

Following recapture, this behavioural structure became less strongly dominated by a single axis, indicating a redistribution of variance across multiple behavioural dimensions. Although most variation still reflected an active versus passive stress-coping style, this axis exhibited significantly lower explanatory power. Moreover, PC2 captured additional residual variation that was less evident during first captures, suggesting increased behavioural differentiation across individuals after recapture events. Overall, these results suggest that previous experience reduces the dominance of a few behaviours (reflecting an active vs passive coping style), in favour of a broader set of behaviours that capture other aspects of individuals' behavioural type.

These interpretations should be considered in light of three important constraints. First, seasonal reproductive condition may contribute to behavioural variation in *A. mystacinus*, as social and agonistic behaviour can vary between reproductive and non-reproductive periods. However, our sampling did not include the summer period described by Váchová & Frynta [79], and most recaptures occurred over short intervals; therefore, the observed capture-history-associated patterns are unlikely to reflect only a broad reproductive versus non-reproductive seasonal contrast. Second, capture itself is not a random process: risk-taking tendencies, microhabitat preferences and individual variation in vigilance or boldness likely influence which animals enter traps (e.g. ‘trap-happy’ animals). For example, in lizards, different trapping methods selectively sample distinct behavioural types, raising the possibility that behavioural studies based on captured animals may not fully represent the true variation present in the underlying population. Because our assessment of behavioural differences was inferred from behaviour expressed during and immediately after entering the box, it is possible that our sample reflects only a subset of the behavioural diversity present in the studied population, as noted elsewhere for small mammals [31,72]. Although previous work suggests that consistent among-individual differences in behaviour might not predict trappability [34], behaviours observed during trapping might themselves represent meaningful behavioural differentiation among individuals. Indeed, this is one of our main findings. In any case, individuals in our study differed in their behaviours before and after exiting the box, indicating that post-capture responses are shaped by the interaction of intrinsic traits and experience. Third, studies like ours, and even more detailed observations of behavioural transitions [80], are important for establishing general principles of how animals respond to handling and repeated capture-related stressors in the wild [8,81]. While we acknowledge that the number of unique individuals caught was relatively low (*n* = 27), the total number of capture events, including recaptures, was 93 observations, giving an average of more than three samples per individual. This sample size allowed us to reliably estimate consistent among-individual variation, as well as changes, in behaviour as a function of body mass, sex and prior experience. As such, our inference is grounded in repeated behavioural sequences rather than cross-sectional comparisons, which increases robustness despite a modest sample size of individuals.

## 5. Conclusions

Taken together, our results show that capture–recapture events are associated with consistent among-individual differences in behavioural responses immediately after release in *A. mystacinus*, while also being modulated by body mass and previous experience with trapping. In other words, behavioural variation in the context of trapping reflects a combination of consistent individual differences and plastic, experience-dependent adjustments that are further mediated by individual condition. By quantifying behaviour as a sequence of behavioural states, rather than as isolated traits, our study demonstrates that sequential behavioural measurements can reveal how experience with a stressful stimulus reshapes the temporal organisation of stress responses. Our findings also suggest that behaviours measured immediately after release may not represent an individual's undisturbed behavioural phenotype, but rather the outcome of an interaction between intrinsic behavioural tendencies and an individual's cumulative experience with previous environmental challenges. More broadly, we highlight the value of fine-scale behavioural data for understanding how animals respond to environmental stressors and for improving the interpretation of behavioural responses in ecological studies. Integrating these behavioural measurements with physiological indicators of stress across repeated encounters will be an important next step towards identifying the proximate mechanisms underlying flexibility in stress-coping strategies in wild populations.

## Supplementary Material



## Data Availability

The dataset generated is available at Zenodo [83]. Supplementary material is available online [84].
