# Supplementary material for: Experience-dependent shifts in behavioural decision-making following repeated captures in a wild mammal
Source: R Soc Open Sci. 2026 Oct 21;13(10):260223. doi: 10.1098/rsos.260223 (PMC13682897; doi:10.1098/rsos.260223)
Supplement: Supplementary file 1 [file rsos.260223.zip › rsos.260223/rsos260223_si_001.docx]

**Supplementary material**

**Experience-dependent shifts in behavioural decision-making following repeated captures in a wild mammal**

**Figure S1**. The Standardized Holding–Release Chamber used. Mice were placed inside and were given the chance to leave. We recorded their behaviour for up to five minutes immediately after we opened all four doors.

| Table S1. Repeatability analysis: R= repeatability estimate, the 95 % confidence interval and the p-value are given for each model. | | | |
| --- | --- | --- | --- |
| *Model* | ***R*** | ***CI*** | ***p*** |
| Model 1: Exited or not the box? | 0.27 | 0.02 to 0.77 | 0.052 |
| Model 2: Time to exit the box | 0.29 | 0.04 to 0.59 | **< 0.05** |
| Model 3: Time spent in any behavioural states | 0.25 | 0.15 to 0.50 | **< 0.001** |

| **Table S2.** Results of a generalized linear mixed model (model 4) and two linear mixed effects models (models 5 & 6) testing if capture number, sex and body weight predict the probability that a mouse exits the box, the latency to first exit and the time allocated across different behavioural states. The estimates, the 95 % confidence interval and the p-value are given for each model. | | | | | | | | | |
| --- | --- | --- | --- | --- | --- | --- | --- | --- | --- |
| ***Predictors*** | **Model 4:**  **Exited or not the box?** | | | **Model 5:**  **Time to exit the box** | | | **Model 6:**  **Time spent in any behavioural state** | | |
|  | ***Log-Odds*** | ***CI*** | ***p*** | ***Estimates*** | ***CI*** | ***p*** | ***Estimates*** | ***CI*** | ***p*** |
| Intercept | 0.45 | -0.56 – 1.5 | 0.38 |  |  |  | -0.52 | -0.88 – -0.17 | **0.004** |
| Capture number | 1.43 | 0.56 – 2.29 | **0.001** | 0.68 | 0.39 – 0.98 | **<0.001** | -0.35 | -0.48 – -0.21 | **<0.001** |
| Body weight | -0.80 | -1.50 – -0.09 | **0.026** | -0.45 | -0.86 – -0.03 | **0.034** | 0.15 | -0.01 – 0.31 | 0.071 |
| Males | 0.39 | -1.03 – 1.80 | 0.592 | 0.46 | -0.47 – 1.38 | 0.336 | -0.20 | -0.61 – 0.22 | 0.349 |
| Frozen outside |  |  |  |  |  |  | 0.55 | 0.16 – 0.94 | **0.006** |
| Inside the box |  |  |  |  |  |  | 1.00 | 0.74 – 1.25 | **<0.001** |

| Table S3. Transition probability matrices (P) derived from the multi-state continuous-time Markov model. The probabilities represent the likelihood that an individual transitions from one behavioural state (rows: “From State”) to another (columns) between two consecutive observed states in the behavioural sequence (i.e. a single step, defined as the change from one recorded behavioural state to the next in time). Results are shown for representative covariate profiles during first captures (First capture = yes) and recaptures First capture = no): lower than average weight (Weight = -1), higher than average weight (Weight = +1), at an average weight (Weight = 0). The "Running" state was modelled as an absorbing state, meaning that once an individual entered this state it could not transition back to any other behavioural state for this specific capture. *No = recapture. | | | | | | |
| --- | --- | --- | --- | --- | --- | --- |
| Weight | **First capture** | **From State** | **Inside** | **Exploring** | **Frozen** | **Running** |
| -1 | Yes | Inside | 0.254 | 0.481 | 0.145 | 0.118 |
| -1 | Yes | Exploring | 0.238 | 0.464 | 0.140 | 0.156 |
| -1 | Yes | Frozen | 0.223 | 0.435 | 0.132 | 0.209 |
| -1 | Yes | Running | 0.000 | 0.000 | 0.000 | 1.000 |
| -1 | No* | Inside | 0.057 | 0.358 | 0.250 | 0.333 |
| -1 | No | Exploring | 0.042 | 0.285 | 0.227 | 0.444 |
| -1 | No | Frozen | 0.034 | 0.168 | 0.146 | 0.649 |
| -1 | No | Running | 0.000 | 0.000 | 0.000 | 1.000 |
| 0 | Yes | Inside | 0.181 | 0.470 | 0.171 | 0.177 |
| 0 | Yes | Exploring | 0.156 | 0.440 | 0.162 | 0.240 |
| 0 | Yes | Frozen | 0.137 | 0.391 | 0.145 | 0.326 |
| 0 | Yes | Running | 0.000 | 0.000 | 0.000 | 1.000 |
| 0 | No | Inside | 0.079 | 0.398 | 0.182 | 0.339 |
| 0 | No | Exploring | 0.057 | 0.319 | 0.162 | 0.459 |
| 0 | No | Frozen | 0.045 | 0.177 | 0.092 | 0.684 |
| 0 | No | Running | 0.000 | 0.000 | 0.000 | 1.000 |
| 1 | Yes | Inside | 0.135 | 0.439 | 0.183 | 0.241 |
| 1 | Yes | Exploring | 0.098 | 0.392 | 0.169 | 0.339 |
| 1 | Yes | Frozen | 0.076 | 0.317 | 0.138 | 0.467 |
| 1 | Yes | Running | 0.000 | 0.000 | 0.000 | 1.000 |
| 1 | No | Inside | 0.101 | 0.438 | 0.121 | 0.338 |
| 1 | No | Exploring | 0.071 | 0.358 | 0.106 | 0.463 |
| 1 | No | Frozen | 0.056 | 0.202 | 0.057 | 0.683 |
| 1 | No | Running | 0.000 | 0.000 | 0.000 | 1.000 |

| Table S4. PCA loadings for first captures | | |
| --- | --- | --- |
| **Variable** | **PC1** | **PC2** |
| Time spent inside the box | 0.553 | -0.123 |
| Time spent exploring outside the box | -0.501 | 0.346 |
| Time spent frozen/stationary outside the box | -0.368 | -0.914 |
| Time to exit the trap | 0.553 | -0.170 |

| Table S5. PCA loadings for recaptures | | |
| --- | --- | --- |
| **Variable** | **PC1** | **PC2** |
| Time spent inside the box | 0.622 | -0.062 |
| Time spent exploring outside the box | -0.306 | -0.775 |
| Time spent frozen/stationary outside the box | -0.337 | 0.628 |
| Time to exit the trap | 0.635 | 0.021 |
